# Beyond Structural Pathology: Central Sensitization and Chronic Pain with Reference to Lumbar Disc Herniation—A Narrative Review

**DOI:** 10.3390/brainsci16070664

**Published:** 2026-06-25

**Authors:** Igor Kordowski, Maciej Chroboczek

**Affiliations:** 1 Faculty of Physiotherapy, Gdansk University of Physical Education and Sport, 80-336 Gdansk, Poland; igorkordowski@gmail.com; 2 Department of Physiology, Gdansk University of Physical Education and Sport, 80-336 Gdansk, Poland

**Keywords:** central sensitization, lumbar disk herniation, nociplastic pain, pain neuroscience education, kinesiophobia, quantitative sensory testing, biopsychosocial model, rehabilitation

## Abstract

**Highlights:**

**What are the main findings?**

- Central sensitization (CS) seems to be an important mechanism for the development of persistent pain and disability in chronic low back pain and lumbar disk herniation, which may explain the absence of a direct relationship between symptom severity and structural findings. However, CS should be seen as a part of a multidimensional biopsychosocial model, not a diagnosis in itself.
- Evidence supports a multimodal approach to management for patients with CS-related symptoms. Pain neuroscience education, exercise-based rehabilitation, psychologically informed interventions, and some adjunctive treatments (e.g., dry needling or centrally acting medications) may improve pain, function, and psychosocial outcomes, especially when used in combination rather than separately.

**What are the implications of the main findings?**

- While clinicians may benefit from pure structural assessment, clinical evaluation frameworks could also be informed by examining central pain processing, psychosocial factors (e.g., kinesiophobia, catastrophizing), and functional limitations through multimodal assessment strategies—such as the CSI (Central Sensitization Inventory), QST (quantitative sensory testing), and CPM (conditioned pain modulation)—in addition to routine clinical examination.
- The management of patients with lumbar disk herniation and persistent pain may benefit from being tailored to the individual with a biopsychosocial focus emphasizing education, graded exercise, and psychological support, while acknowledging that increased central sensitization does not inherently correlate with a poor prognosis, including the potential for surgical resolution.

**Abstract:**

Chronic pain is increasingly understood as a multidimensional condition in which, in a substantial subgroup of patients, a protective symptom can evolve into a persistent maladaptive disorder of the nervous system, while in others it may remain closely tied to ongoing mechanical or structural factors. Central sensitization (CS) represents a key mechanism underlying this transition, characterized by enhanced neural responsiveness and impaired endogenous pain inhibition, leading to a dissociation between pain and tissue pathology. The aim of this narrative review is to critically discuss current evidence on CS as a mechanism-based explanation for persistent pain, using lumbar disk herniation (LDH) as a clinical model of the radiological-clinical mismatch, and to discuss its direct implications for identifying sensitized phenotypes, multimodal assessment, and rehabilitation strategies. A total of 77 sources published between 2006 and 2026 were synthesized. These reviewed sources demonstrate that identification of the sensitized phenotype requires a multimodal assessment approach combining self-report measures, such as the Central Sensitization Inventory (CSI), with psychophysical methods including quantitative sensory testing (QST) and conditioned pain modulation (CPM). Cognitive-emotional factors are also critical, as postoperative kinesiophobia affects approximately 38.3% of LDH patients and is associated with increased pain intensity and reduced self-efficacy. Management strategies reported in these publications focus on mechanism-based interventions, particularly pain neuroscience education (PNE) and graded, time-contingent exercise, which aim to modify pain-related cognitions and restore endogenous inhibitory processes. These approaches may be supported by adjunctive therapies, including dry needling (DN), electro-dry needling (EDN), centrally acting pharmacological agents (e.g., serotonin–norepinephrine reuptake inhibitors [SNRIs] and gabapentinoids), and psychologically informed treatments such as cognitive behavioral therapy (CBT). While surgical decompression may reduce CS-related symptoms, preoperative sensitization does not necessarily predict poorer outcomes, highlighting the interaction between peripheral and central mechanisms. Adopting a sensitization-informed perspective may encourage a broader integration of contemporary pain models alongside traditional structural views in lumbar disc herniation clinical care.

## 1. Introduction

Chronic pain is increasingly recognized not merely as a prolonged symptom, but as a multidimensional disease state associated with substantial individual, social, and economic burden [1,2]. According to the International Association for the Study of Pain (IASP), pain is defined as an unpleasant sensory and emotional experience associated with, or resembling that associated with, actual or potential tissue damage [1]. While acute pain typically serves a protective biological function and supports tissue healing, pain that persists beyond the expected period of recovery often reflects maladaptive neurobiological processes involving altered nociceptive signaling, psychological distress, and functional impairment [2].

Contemporary pain models increasingly emphasize mechanism-based classification systems that extend beyond traditional structural diagnoses. Pain is commonly categorized as nociceptive, neuropathic, or nociplastic, although many chronic musculoskeletal disorders demonstrate overlapping or mixed pain phenotypes [3,4,5]. Nociceptive pain is associated with activation of peripheral nociceptors in response to tissue injury or inflammation, whereas neuropathic pain arises from lesion or disease of the somatosensory nervous system [3]. Nociplastic pain, in contrast, refers to pain arising from altered nociceptive processing despite the absence of clear ongoing tissue damage or definable somatosensory pathology [4]. Recognition of mixed pain presentations is particularly relevant in spinal disorders, where structural, inflammatory, neuropathic, and centrally mediated mechanisms may coexist [5,6].

Low back pain remains the leading cause of disability worldwide and represents a major contributor to years lived with disability across all age groups [7,8,9]. Recent projections suggest that the global number of individuals living with low back pain may reach approximately 843 million by 2050, largely driven by population aging and demographic growth [7,9]. Importantly, a substantial proportion of this burden appears attributable to modifiable risk factors, including occupational ergonomic exposure, smoking, and high body mass index [8,9].

Within this spectrum, lumbar disc herniation (LDH) is one of the most common and clinically relevant spinal disorders, frequently associated with radicular pain, functional limitation, and healthcare utilization [10,11]. The risk of LDH is highest in adults aged 30–50 years and is strongly associated with cumulative lumbar loading, including repetitive bending, lifting, and occupational mechanical stress [11].

However, structural pathology alone does not fully explain the clinical presentation. While lumbar disc herniation is generally a self-limiting condition with clinical symptom resolution reported in 60–80% of patients within 6–12 weeks, rising to 80–90% over the long term [12], a substantial proportion of patients continue to experience persistent pain and disability despite radiological improvement [13,14]. Notably, approximately 30% of patients managed conservatively still report intermittent pain one year after symptom onset, highlighting the potential for chronification [12].

Genetic susceptibility also influences this trajectory; a variant at 8q24.21 is associated with an increased risk of persistent sciatica requiring surgery, particularly in younger patients [15]. From a clinical standpoint, this variant is particularly significant as it does not dictate the structural morphology of disc herniation but instead alters the individual’s threshold for developing severe, persistent neuropathic pain following nerve root insult. Identifying these genetic markers holds promise as a prognostic strategy to screen for patients predisposed to conservative treatment failure. Consequently, this provides a biological explanation for the divergence between spontaneous recovery and chronic symptom persistence, potentially facilitating early, personalized surgical or neuromodulatory profiling [15]. This persistence is further linked to an altered immune microenvironment, where increased macrophage infiltration and the upregulation of hub genes like ID1 and RAP2C drive the inflammatory signaling that maintains pain [16]. Importantly, postoperative improvement after lumbar discectomy may occur independently of preoperative sensitization status, while central sensitization-related symptoms can also decrease following decompression, suggesting a dynamic interaction between peripheral pathology and central pain modulation [17].

One increasingly discussed explanatory framework is central sensitization (CS), broadly understood as an amplification of neural signaling within the central nervous system leading to pain hypersensitivity, reduced thresholds, and altered endogenous pain modulation [18]. Clinical features associated with CS may include hyperalgesia, allodynia, widespread pain sensitivity, fatigue, sleep disturbance, and disproportionate pain responses relative to peripheral findings [19,20]. Rather than operating as isolated conditions, lumbar pathology and CS share deeply overlapping neurobiological pathways [21]. Persistent nociceptive input and inflammation from damaged lumbar structures act as the primary peripheral trigger, driving a cascade of central alterations that include dorsal horn hyperexcitability, maladaptive neuroplasticity, and cortical remodeling [21]. This transition from localized tissue damage to a generalized nociplastic state underscores how peripheral drivers and central pain amplification actively reinforce each other [21]. In chronic low back pain and related musculoskeletal conditions, central sensitization has been associated with greater pain intensity, disability, and poorer treatment response [19,22]. Notably, depressive symptoms have been reported as one of the strongest independent predictors of sensitization severity, further supporting the multidimensional nature of chronic pain presentations [22].

At the same time, the conceptual and clinical boundaries of central sensitization remain debated. While many authors consider CS a clinically meaningful mechanism-based construct, others argue that its diagnostic operationalization remains inconsistent and that the concept may be overgeneralized across heterogeneous chronic pain populations [23,24,25]. Critically, several commonly used clinical proxies of CS, such as self-report inventories or psychophysical findings, may also reflect broader psychological distress, pain-related behaviors, or non-specific symptom burden rather than direct neurophysiological confirmation of sensitization [24]. Furthermore, recent perspectives advocate distinguishing between the provoking procedure, the observed behavioral response, and the presumed neural mechanism of sensitization in order to reduce explanatory circularity in chronic pain research [25]. These differing perspectives highlight the need for cautious interpretation and for clinically integrated syntheses that distinguish theoretical relevance from practical application.

Despite growing interest in central sensitization, evidence relevant to lumbar disc herniation remains dispersed across the neurophysiological, diagnostic, psychological, and rehabilitation literature. Importantly, parsing spinal disorder heterogeneity requires separating localized structural conditions from presentations driven by altered central mechanisms [21]. Recent simultaneous PET-fMRI neuroimaging has unveiled these central neuroimmune signatures. Individuals with predominant nociplastic low back pain exhibit significantly greater neuroinflammatory and glial activation somatopically localized to the lower back representation within the primary somatosensory and motor cortices (S1/M1) [21,26]. This localized cortical neuroinflammation directly correlates with clinical symptoms of central sensitization, psychological distress, and increased psychophysical pain sensitivity via quantitative sensory testing (QST; a protocol further detailed in subsequent sections of this review) [26]. Aligning these psychophysical and neuroimmune profiles with structural clinical pathways is essential to establish a precise framework specific to lumbar disk pathology, helping clinicians better understand persistent pain presentations insufficiently explained by structural findings alone.

Therefore, the aim of this narrative review was to critically discuss current evidence regarding the role of central sensitization in chronic pain, with reference to lumbar disc herniation, and to provide a comprehensive overview of its implications for multimodal assessment and rehabilitation.

## 2. Methods

### 2.1. Study Design

This study was conducted as a narrative review aimed at critically synthesizing current evidence on central sensitization in chronic pain, with particular reference to lumbar disc herniation and its implications for multimodal assessment and rehabilitation. A narrative review design was considered appropriate because the topic spans heterogeneous domains, including neurophysiology, pain mechanisms, clinical assessment, psychological correlates, and rehabilitation-oriented management, which are difficult to integrate within the stricter methodological framework of a systematic review.

### 2.2. Literature Search Strategy

The literature search was conducted using major biomedical and multidisciplinary databases, including PubMed, ScienceDirect, Google Scholar, and Web of Science. In addition, relevant publications were identified through manual screening of reference lists and searches of topic-specific journals related to pain science, musculoskeletal rehabilitation, physiotherapy, spine disorders, and clinical neuroscience.

The search covered publications published between 2006 and 2026. This time frame was selected to capture both foundational conceptual work on central sensitization and more recent developments in diagnostic approaches, mechanistic understanding, and rehabilitation strategies. Search terms were combined using Boolean operators and adapted to the syntax of each database. The main search terms included combinations of the following keywords: chronic pain, low back pain, lumbar disc herniation, radiculopathy, central sensitization, nociplastic pain, neuropathic pain, mixed pain, quantitative sensory testing, conditioned pain modulation, Central Sensitization Inventory, kinesiophobia, pain catastrophizing, pain neuroscience education, exercise therapy, dry needling, cognitive behavioral therapy, acceptance and commitment therapy, and rehabilitation.

### 2.3. Eligibility and Selection of Sources

Publications were considered eligible if they contributed to at least one of the following domains:

- Conceptual or mechanistic understanding of central sensitization;
- Chronic low back pain or lumbar disc herniation as clinical models of persistent pain;
- Diagnostic and assessment approaches relevant to central pain processing;
- Psychosocial factors associated with sensitization-related symptom profiles;
- Rehabilitation and treatment strategies targeting central sensitization-related mechanisms.

The review included original research studies, systematic reviews, meta-analyses, narrative reviews, clinical recommendations, and selected conceptual papers when they were considered important for interpreting the multidimensional nature of central sensitization. Priority was given to higher-level evidence, including systematic reviews, meta-analyses, clinical guidelines, and well-designed clinical or observational studies. Animal and experimental studies were included selectively when they provided relevant mechanistic insight, particularly in relation to lumbar disc pathology and central pain amplification.

Publications in English and Polish were considered eligible. Sources were excluded if they were clearly unrelated to the focus of the review, addressed pain conditions without relevance to the conceptual framework of central sensitization, or did not contribute meaningful clinical, mechanistic, or rehabilitation-oriented information.

### 2.4. Data Extraction and Synthesis

A total of 77 publications were included in the final review. Given the narrative design, no formal quantitative synthesis or meta-analysis was performed. Instead, the literature was analyzed qualitatively and synthesized thematically. The material was organized into the following major domains:

- Chronic pain and mechanism-based pain classification;
- Epidemiology and clinical relevance of low back pain and lumbar disk herniation;
- Neurophysiological and clinical aspects of central sensitization;
- Assessment tools relevant to central sensitization;
- Psychological and behavioral contributors to pain persistence;
- Central sensitization-informed management strategies, including education, exercise, psychologically informed care, dry needling, pharmacological support, and clinical implications for lumbar disc herniation.

Emphasis was placed on identifying converging and conflicting findings across studies, highlighting clinically relevant patterns, and distinguishing between evidence derived directly from lumbar disc herniation populations and evidence extrapolated from broader chronic pain or nociplastic pain populations.

### 2.5. Methodological Considerations

Because this article was designed as a narrative review, no formal risk-of-bias assessment tool was applied, and the study selection process was not conducted according to systematic review reporting standards. This should be considered when interpreting the findings. Nevertheless, an effort was made to improve transparency and scientific rigor by relying primarily on the peer-reviewed literature, prioritizing higher-quality evidence where available, and integrating both supportive and critical perspectives on the concept of central sensitization.

To assist the reader in distinguishing between the varying levels of evidence, a hierarchical approach was applied to the text. Major clinical and therapeutic claims are anchored in systematic reviews and meta-analyses (with specific design characteristics highlighted in the text where relevant), while conceptual frameworks and neurobiological pathways rely on observational data or foundational models.

## 3. Central Sensitization

**Figure 1:**
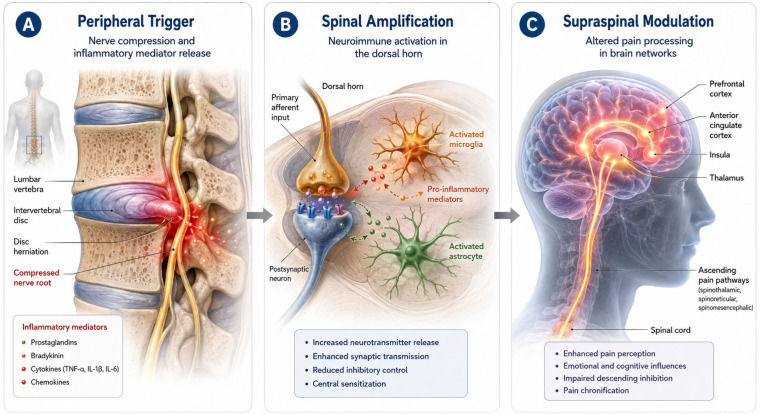
The neurobiological continuum of pain chronification in lumbar disc herniation. (**A**) Peripheral trigger: Mechanical compression of the nerve root by herniated disc material initiates the local release of pro-inflammatory mediators (prostaglandins, bradykinin, chemokines, and **cytokines** such as TNF-alpha, IL-1 beta, and IL-6). (**B**) Spinal Amplification: Persistent primary afferent input triggers neuroimmune activation within the dorsal horn, characterized by microglia and astrocyte activation, increased neurotransmitter release, and enhanced synaptic transmission, leading to central sensitization and reduced inhibitory control. (**C**) Supraspinal Modulation: Chronic ascending pain pathways alter centralized processing within higher brain networks (including the prefrontal cortex, anterior cingulate cortex, insula, and thalamus), resulting in enhanced pain perception, cognitive-emotional modifiers, and impaired descending inhibition.

Although introduced conceptually in the preceding section, central sensitization (CS) warrants deeper mechanistic consideration due to its growing relevance in chronic spinal pain. Experimental and clinical evidence suggests that CS involves maladaptive neuroplastic changes within spinal and supraspinal nociceptive pathways, resulting in enhanced pain facilitation, impaired inhibitory control, and increased responsiveness to sensory input [18,20]. The transition from localized mechanical compression to generalized central hypersensitivity involves a multi-tiered neurobiological cascade encompassing peripheral triggers, spinal amplification, and supraspinal networks (Figure 1).

Recent evidence suggests that CS may serve as a potential explanatory framework for the frequent mismatch between structural pathology and pain severity in chronic spinal disorders [21]. To bridge the gap between clinical observations of this radiological-clinical mismatch and underlying neurobiology, preclinical animal models provide essential insights regarding biological plausibility, illustrating how localized disc pathomechanics and chemical radiculitis drive subsequent spinal hyperexcitability [21,27]. However, these experimental models of lumbar disc herniation represent mechanistic inferences rather than direct clinical evidence. For instance, in a rodent model using nucleus pulposus implantation, persistent mechanical allodynia and thermal hyperalgesia were accompanied by increased synaptic transmission efficiency in the spinal dorsal horn, microglial activation, and upregulation of pro-inflammatory cytokines [27]. While these hallmark features of CS offer a plausible translational explanation for radicular pain, caution is traditionally warranted when extrapolating these laboratory data directly to human LDH cohorts. Importantly, recent comprehensive reviews support this translational link, demonstrating that similar neuroimmune crosstalk—such as elevated pro-inflammatory cytokines (e.g., IL-8) in the cerebrospinal fluid—is also present in patients with chronic LDH, directly correlating with pain severity and central hyperalgesia [21].

Mechanistically, CS is associated with measurable neurobiological alterations at both spinal and supraspinal levels. Neuroimaging studies in patients with chronic low back pain and lumbar disc herniation demonstrate abnormal activity in brain regions involved in pain modulation, emotional regulation, and cognitive control, including prefrontal and temporal cortices, with these alterations correlating with pain intensity and psychological distress [28]. Furthermore, psychophysical findings indicate that these alterations are manifested as sensory deficits; patients with a pronounced neuropathic component exhibit elevated tactile detection thresholds (TTDs), while two-point discrimination remains preserved. This pattern suggests maladaptive supraspinal neuroplastic changes within the somatosensory cortex rather than isolated peripheral nerve compression. These sensory deficits correlate with significantly higher levels of pain intensity, functional disability, and psychological distress [29]. In parallel, experimental and clinical evidence indicate that CS is accompanied by neuroimmune activation, reflected by increased expression of pro-inflammatory cytokines and microglial activity within the spinal cord and central nervous system, further supporting the biological plausibility of sustained central pain amplification [23].

A comprehensive review demonstrated that features of CS are consistently present in chronic low back pain and are associated with poorer treatment outcomes, emphasizing the need for pain phenotyping beyond structure-based classification [19]. Clinical evidence confirms that in patients with chronic musculoskeletal pain, particularly chronic low back pain, CS manifests as lowered pain thresholds, sensory hypersensitivity, sleep disturbances, fatigue, and poorer response to treatments targeting peripheral tissues [30]. From a physiotherapy perspective, this symptom profile underscores the clinical relevance of CS as a distinct pain mechanism requiring approaches beyond structural or tissue-focused interventions [31].

To objectively identify this phenotype, researchers and clinicians increasingly rely on assessment tools such as quantitative sensory testing (QST), conditioned pain modulation (CPM), and the Central Sensitization Inventory (CSI). Currently, these instruments are among the most consistently utilized measures of CS in research and clinical practice [32], and their application is supported by extensive psychometric validation and reliability studies [33,34,35,36,37].

In routine clinical practice, the feasibility and practical implementation of these assessment modalities vary significantly. Laboratory-grade QST and CPM protocols require sophisticated, calibrated thermal or mechanical stimulators and rigorous, standardized examiner training to ensure intra- and inter-rater reliability. Consequently, these psychophysical methods remain primary fixtures of specialized multidisciplinary pain clinics and research units rather than primary care environments [19]. Conversely, the CSI represents a highly accessible, zero-cost, self-report instrument that requires no specialized equipment or formal certification. The CSI can be seamlessly implemented by frontline physical therapists in everyday clinical settings as an initial screening proxy to quantify central symptom burden and guide mechanism-informed clinical decision-making.

Complementary self-report instruments, such as the Pain Catastrophizing Scale (PCS) and Tampa Scale for Kinesiophobia (TSK), are also commonly included to assess cognitive-emotional and behavioral factors associated with central sensitization severity and functional impact [38,39,40,41].

Together, these findings demonstrate that CS is a clinically relevant mechanism-based construct that may influence not only how pain is generated, but also how it persists and responds to treatment. At the same time, recent data show a lack of conclusive evidence that CS causes chronic pain. CS is strictly a neurophysiological phenomenon requiring direct measurement (e.g., EEG, fMRI) rather than inference from reported symptoms. Furthermore, a pervasive methodological flaw in current research involves confounding subjective pain assessments—such as QST or CSI—with actual measurements of nervous system sensitization. These clinical tools identify specific pain phenotypes, not the neurophysiological occurrence of CS itself. Since there is currently no direct evidence proving that CS causes chronic pain, or even definitively documenting its isolated presence in humans, establishing CS as a standalone clinical diagnosis may be premature based on the currently available evidence [19,24,25].

## 4. Management of Central Sensitization

Managing CS requires a multidimensional strategy that targets both altered neurophysiological pain processing and the cognitive, emotional, and behavioral factors that contribute to pain persistence [42]. Individuals with chronic low back pain frequently display elevated CS levels, which are associated with higher pain intensity, greater disability, and reduced quality of life [30,34,36]. Psychological factors, including fear of movement, catastrophizing, anxiety, and depressive symptoms, appear to play an important role in amplifying central sensitization, as they may increase forebrain involvement in pain processing and contribute to the maintenance of central hyperexcitability [22,30].

Recent evidence indicates that greater central sensitization severity is independently associated with higher levels of kinesiophobia, potentially contributing to a self-perpetuating cycle of movement avoidance, deconditioning, and disability [39,40]. In parallel, depression has been identified as the strongest independent predictor of sensitization severity in chronic musculoskeletal pain populations [22]. Collectively, these findings support the use of multidimensional management strategies that address both altered central pain processing and key psychosocial contributors through combinations of exercise, education, and psychologically informed interventions [19,43,44].

It should be noted that while the therapeutic frameworks discussed in this section are conceptualized with reference to lumbar disc herniation, much of the underlying clinical evidence originates from broader chronic musculoskeletal pain populations, non-specific low back pain, or mixed chronic pain cohorts, from which mechanistic principles are extrapolated.

### 4.1. Pain Neuroscience Education

Pain neuroscience education (PNE) is considered a foundational intervention for patients presenting with features of central sensitization (CS). It is an educational model designed to teach patients about the complex biology and physiology of their pain experience [43,45]. PNE reframes pain as a manifestation of altered nociceptive processing and nervous system hypersensitivity rather than a direct indicator of structural tissue damage [20,45,46]. However, PNE may be particularly useful when individualized to the patient’s specific pain presentation [19]. In patients with central sensitization, education should help explain altered nociceptive processing and nervous system hypersensitivity, while also acknowledging that structural, nociceptive, or fear-related contributors may coexist and influence the pain experience [19]. This balanced reconceptualization is clinically important, as traditional biomedical models focused solely on “damaged tissues” may inadvertently increase fear, anxiety, and catastrophic thinking, thereby reinforcing central hyperexcitability [20,45].

Accumulating evidence from systematic reviews indicates that PNE is associated with reductions in pain intensity, disability, kinesiophobia, and pain catastrophizing, particularly when integrated into active rehabilitation programs [45,47,48].

However, the quality of this evidence varies. For instance, one review of 8 trials (*n* = 615) noted that the studies were highly diverse and reported low-quality evidence for PNE alone, though moderate-quality evidence supported it as an adjunct to physical therapy [48]. Due to high study diversity, other reviews—one analyzing 13 trials (*n* = 734) [43] and another analyzing 15 trials (*n* = 1085) [47]—could not combine their data into a meta-analysis and relied only on qualitative descriptions. Furthermore, while the 13 trials in one review showed good methodological quality [45], the other review highlighted a high risk of performance bias across all 15 included trials [47]. Consequently, while PNE remains a clinically valuable tool, its effects appear more modest and highly variable when delivered as a standalone intervention rather than alongside exercise-based treatment [47,48].

Nevertheless, brief internet-based PNE interventions can yield significant short-term reductions in pain severity and interference, particularly for patients with localized spinal pain. By encouraging patients to reinterpret pain as a “false alarm” rather than ongoing tissue damage, these modalities facilitate a critical shift from “peripheral injury” to “brain-based” attributions [49].

Importantly, PNE should be viewed as a preparatory intervention that reduces perceived threat and symptom-driven avoidance [46]. By helping patients understand pain through a biopsychosocial lens, PNE may facilitate earlier engagement in graded, time-contingent exercise and support modulation of central pain pathways [45,50]. High-level evidence further suggests that outcomes improve when active rehabilitation strategies are combined with PNE, as education can reduce affective–emotional barriers to movement [47,51].

### 4.2. Exercise-Based Interventions

Exercise-based interventions are thought to modulate dysfunctional central pain regulatory mechanisms [41,48]. Neuroimaging evidence from patients with fibromyalgia, a prototypical nociplastic pain condition, suggests that moderate-intensity exercise may reduce pain sensitivity while enhancing activation of descending inhibitory networks, particularly in the left dorsolateral prefrontal cortex (DLPFC) and anterior insula, regions involved in endogenous pain modulation [52]. These findings indicate that exercise may transiently normalize central pain modulation and improve the functional capacity of the modulatory system rather than acting solely through peripheral or functional mechanisms [50,52].

From a clinical perspective, individuals with CS frequently exhibit a blunted or even reversed exercise-induced hypoalgesic (EIH) response, where pain sensitivity remains unchanged or increases following activity [46,50,53].

Despite these acute flares, limited meta-analytical evidence suggests that consistent exercise training may increase pressure pain thresholds, potentially offering improvements in pain sensitivity compared to passive interventions such as massage or education alone, although the overall certainty of this evidence remains low to very low [54]. This aligns with preliminary findings indicating a potential link between regular physical activity and altered pain perception, as physically active cohorts tend to exhibit higher pressure pain thresholds and tolerance compared to inactive counterparts; however, these quantitative effects are often modest and highly dependent on exercise parameters and individual patient clinical profiles [19,55].

A network meta-analysis pooling data from 89 RCTs (*n* = 6223) suggests that multi-component exercise—particularly the combination of strengthening and stretching—presents a favorable strategy for reducing CS indices [56]. However, these findings require cautious interpretation due to a very high study diversity of 89% and low certainty of evidence according to GRADE assessments [56]. Furthermore, a systematic review of 14 RCTs (*n* = 777) noted that for patients with more severe symptoms, moderate-intensity water-based aerobic exercise (e.g., at least 60 min, 1–2 times weekly) could provide short-term pain relief [57]. While this review reported moderate overall certainty of evidence, it also highlighted a substantial statistical diversity of 86.4% across the primary studies [57].

More broadly, based on a systematic review encompassing 26 RCTs (*n* = 1649) focused on conditions with a predominance of nociplastic pain, current recommendations support supervised global exercise sessions lasting 50–60 min, performed 2–3 times per week for at least 13 weeks [58]. However, the authors caution that these parameters are derived from very low- to low-quality evidence according to GRADE criteria, driven primarily by great methodological diversity and small sample sizes across the included studies [58].

From a clinical perspective, these findings suggest tailoring the exercise prescription to the patient’s specific clinical presentation: multi-component routines (strengthening and stretching) are optimal for directly targeting central sensitization indices [56], water-based aerobic exercises are particularly suited for immediate relief in patients with severe pain [57], while long-term, supervised global exercise programs are recommended for managing broad nociplastic pain conditions [58].

Consequently, graded activity and graded exposure approaches are commonly applied to incrementally reintroduce movement while minimizing perceived threat and avoiding symptom exacerbation [43,50]. Practically, this involves “exposure with control”—systematically reintroducing feared movements in a safe environment. During these tasks, clinicians can use Socratic-style dialog to help patients reframe exercise-induced pain flares. By explaining that temporary hyperalgesia does not equal new tissue damage, clinicians reduce the threat value of pain, improving patient acceptance and confidence to progress with rehabilitation [43,50]. Crucially, exercise-based interventions must be strictly individualized and progressively adapted to each patient’s specific clinical presentation, needs, and functional goals. Rather than simply increasing general physical activity, prescribing rehabilitation requires precise tailoring of exercise parameters, including intensity, repetitions, progression, and the specific type of movement. Inadequate tailoring risks worsening clinical pain, increasing patient discouragement, and reinforcing fear of movement. Therefore, trained rehabilitation professionals play an essential role in evaluating complex clinical factors—such as kinesiophobia, multiple pain sites, and coexisting radicular symptoms—to safely support individuals living with chronic pain and central sensitization. These strategies should follow a time-contingent approach (e.g., performing exercise for a set duration regardless of pain), which may help promote adaptive learning, reduce top-down pain facilitation, and decrease kinesiophobia [20]. High-level evidence further indicates that outcomes are improved when active interventions are combined with pain neuroscience education (PNE), as education can help reconceptualize pain and reduce affective–emotional barriers to movement [47,51].

### 4.3. Psychological and Cognitive-Behavioral Interventions

Psychological interventions—including cognitive-behavioral therapy (CBT), acceptance and commitment therapy (ACT), mindfulness-based strategies, and relaxation techniques—address the cognitive and emotional mechanisms that sustain CS [20,46,59]. These approaches aim to improve voluntary control over top-down pain facilitatory pathways, targeting the cognitive-emotional processes (e.g., pain catastrophizing, anxiety, depression, kinesiophobia, hypervigilance) that may contribute to symptom persistence [46,59]. The implementation of these strategies can be further enhanced by the clinical use of metaphors, which serve as essential tools for helping patients understand their pain experience and facilitate the re-conceptualization of pain as a protective alarm system rather than a marker of permanent tissue damage [60]. Cognitive-behavioral therapy (CBT) focuses on restructuring maladaptive beliefs, attitudes, and behaviors, which may reduce overall symptom burden by helping patients reinterpret negative thoughts and pain-related beliefs [2,20]. Acceptance and commitment therapy (ACT) specifically targets psychological flexibility, encouraging patients to accept the presence of pain without excessive avoidance and to engage in valued activities, an approach shown to mediate reductions in disability and depression [20,59]. Furthermore, stress-management strategies such as mindfulness may promote adaptive neuroplasticity and reduce sympathetic arousal, potentially decreasing activity in brain regions involved in pain anticipation and emotional evaluation [20].

Recent clinical evidence highlights the important role of psychological factors in patients with LDH. In a retrospective study of 342 postoperative individuals, kinesiophobia—a common feature of CS—was present in 38.3% of cases and showed strong associations with higher pain intensity, anxiety, depressive symptoms, and reduced self-efficacy [61]. Crucially, predictive decision-tree analysis highlighted self-efficacy as the pivotal factor determining the emergence of fear-avoidance patterns. By allowing individuals to constructively reframe pain, sustain recovery optimism, and modulate distressing emotions, strong self-efficacy functions as an essential psychological safeguard. Consequently, rather than being a mere baseline characteristic, self-efficacy operates as a vital mediator of postoperative success, emphasizing the clinical value of proactive, targeted strategies aimed at bolstering a patient’s perceived capability to optimize rehabilitative outcomes [61]. These findings are supported by evidence that depression is the strongest independent predictor of sensitization severity in chronic low back pain populations [22].

While fibromyalgia and LDH represent fundamentally distinct clinical contexts—the former being a widespread nociplastic condition and the latter characterized by localized mechanical and neuro-inflammatory radicular processes—evidence from prototypical central sensitization populations offers a valuable conceptual framework for understanding these psychological dimensions. Consistent with these findings, recent evidence in fibromyalgia, a prototypical central sensitization condition, indicates that anxiety and pain acceptance significantly mediate the relationship between post-traumatic stress symptoms and central sensitization severity [62]. This association may reflect disturbances in affect regulation, characterized by heightened threat-related responses and reduced calming or self-soothing processes, which may maintain increased salience and symptom vigilance [62]. Qualitative research further suggests that psychologically informed and pain-focused educational interventions can enhance self-efficacy, reduce perceived threat, and facilitate re-engagement in valued activities [63]. Such changes may help patients reconceptualize fibromyalgia from a progressive or disabling condition to one that can be more effectively self-managed [63]. Nevertheless, because of these distinct clinical profiles, extrapolating the efficacy of these psychological interventions from a widespread nociplastic condition directly to structurally driven LDH cohorts introduces a degree of uncertainty that must be recognized.

### 4.4. Dry Needling and Electro-Dry Needling

Dry needling (DN), including electro-dry needling (EDN), has been investigated as a useful adjunct intervention for patients with features of altered central pain processing [60,64]. DN involves the insertion of solid filiform needles directly into targeted muscle tissue, utilizing either dynamic manipulation or static placement, to inactivate myofascial trigger points and mitigate peripheral sources of nociception. EDN advances this technique by using the inserted needles as internal electrodes connected to a high-frequency electrical current, thereby bypassing skin resistance to deliver deep and highly effective intramuscular stimulation [64,65]. Clinical evidence indicates that these approaches are associated with significant improvements in Central Sensitization Inventory (CSI) scores and other sensitization-related outcomes, suggesting a meaningful reduction in symptom burden [64,65]. In particular, EDN has shown greater effectiveness than conventional trigger point DN in reducing CSI and pain scores in patients with lumbosacral radiculopathy, potentially due to more effective intramuscular electrical stimulation.

Although applied peripherally, the effects of DN are thought to involve modulation of nociceptive input with secondary influences on central pain processing [46,65]. Myofascial trigger points may act as peripheral drivers of central sensitization, and their inactivation through needling may reduce ongoing nociceptive input to the spinal cord, thereby attenuating central hyperexcitability. Neurophysiologically, DN may activate Aβ and Aδ fibers, promoting segmental inhibition (gate control) and release of endogenous opioids, serotonin, and catecholamines involved in pain modulation [46,65]. Recent evidence further suggests that at least five treatment sessions may be required to induce more sustained neuroplastic changes [65].

Despite these potential benefits, current clinical practice guidelines provide only a Grade C recommendation for DN [43], indicating that it should be used alongside other active treatments to reduce pain and disability in the short term [43]. Moreover, its effects may be enhanced when combined with pain neuroscience education (PNE), which can help patients interpret needling-related sensations as therapeutic rather than threatening stimuli [47,66]. Consequently, DN may serve as a complementary element of multimodal rehabilitation by reducing symptom interference and facilitating earlier engagement in physical activity [46,65].

### 4.5. Pharmacological Approaches

Pharmacological treatment plays a supportive role in the management of central sensitization (CS), particularly when symptoms are pronounced and interfere with the implementation of active rehabilitation strategies [20,44]. Centrally acting medications, such as serotonin-norepinephrine reuptake inhibitors (SNRIs), pregabalin, gabapentin, and low-dose tricyclic antidepressants (TCAs), may be more appropriate than peripherally targeted treatments (e.g., NSAIDs) in individuals with sensitization-related pain profiles, as conventional anti-inflammatory drugs often provide limited benefit in nociplastic pain conditions [2,44]. These agents modulate neurotransmitter systems involved in central pain amplification and should be used to complement, rather than replace, non-pharmacological interventions such as exercise and education [2,44].

SNRIs (e.g., duloxetine) and TCAs (e.g., amitriptyline) increase the availability of serotonin and norepinephrine, neurotransmitters involved in descending inhibitory pain pathways, and may thereby improve conditioned pain modulation [44,46]. Gabapentinoids bind to the α2δ subunit of voltage-gated calcium channels, reducing the release of excitatory neurotransmitters such as glutamate and substance P within the spinal dorsal horn, which may help dampen central hyperexcitability [44,46]. Emerging evidence also highlights tapentadol, a dual-mechanism agent combining μ-opioid receptor agonism with norepinephrine reuptake inhibition (MOR-NRI), which may help limit pain chronification through enhancement of descending inhibition [44,46].

Notably, centrally acting agents may be particularly valuable in the early phases of rehabilitation by helping restore impaired exercise-induced hypoalgesia (EIH) and reducing the likelihood of post-exercise pain flares, which may improve adherence to active treatment protocols [50]. Ultimately, an individualized pharmacological approach targeting central pain mechanisms may also help manage comorbid depression, which has been identified as a strong independent predictor of sensitization severity in chronic low back pain populations [22].

### 4.6. Clinical Implications in Lumbar Disc Herniation

Patients with lumbar disc herniation (LDH) who exhibit higher preoperative central sensitization (CS) often report greater baseline pain intensity and functional disability [17,22]. However, current evidence suggests that postoperative outcomes, including pain relief and functional recovery, are not solely determined by preoperative sensitization levels measured with the Central Sensitization Inventory (CSI) [17]. Importantly, surgical decompression has been associated with significant reductions in CSI scores at 12-month follow-up, possibly through reduction in ongoing nociceptive input and improvement of the local inflammatory environment around affected nerve roots [17]. These findings suggest that patients with elevated CS may still benefit from discectomy, although their recovery pathway may be more complex because of greater initial symptom burden [10,17].

The persistence of symptoms in some patients underscores the need to incorporate CS-oriented strategies throughout the perioperative period. Postoperative kinesiophobia remains an important barrier to recovery, showing strong associations with higher pain intensity and lower self-efficacy [61]. Within this context, non-surgical rehabilitation approaches, including pain neuroscience education (PNE), psychological support, and time-contingent exercise-based retraining, are strongly encouraged [43,67].

However, it is crucial to acknowledge that the direct evidence base for these CS-targeted interventions, specifically in LDH populations, remains limited. Much of the current rationale is extrapolated from studies on fibromyalgia and non-specific chronic low back pain, where these therapies have shown efficacy in addressing cognitive-emotional influences and ongoing neural sensitization [19,45,46,47,48]. Therefore, while these interventions represent promising first-line strategies for managing persistent pain in LDH, their specific impact on LDH outcomes requires further targeted validation (Table 1).

## 5. Discussion

This narrative review aimed to synthesize and critically evaluate current evidence on central sensitization (CS) in chronic pain, with reference to lumbar disk herniation (LDH), and to translate these findings into clinically meaningful diagnostic and therapeutic frameworks. The available evidence suggests that CS represents an important mechanistic component of persistent pain; however, its role should be interpreted within a broader, multidimensional model rather than as a standalone explanatory construct [19,23,68].

Although central sensitization is increasingly used to explain the discrepancy between structural pathology and symptom severity, this relationship remains complex and not fully specific [19]. The frequently observed clinical–radiological mismatch in LDH supports the need for mechanisms beyond purely structural explanations [14,28], yet it does not constitute direct evidence of CS [17]. Persistent symptoms may instead reflect a dynamic interaction between peripheral nociceptive input, neuropathic processes, inflammatory activity, and central modulation [3,4,27,69]. This interpretation aligns with emerging perspectives emphasizing that chronic pain phenotypes are often mixed rather than attributable to a single dominant mechanism [5,30].

Importantly, the conceptual and clinical utility of central sensitization remains a subject of ongoing debate. While a substantial body of the literature supports central sensitization as a meaningful framework for understanding chronic pain [18,19], conflicting perspectives have also been reported. Some authors argue that the construct lacks sufficient conceptual clarity and may be overgeneralized across heterogeneous patient populations [24,25], which limits its direct clinical applicability and highlights the need for further refinement and empirical validation. These discrepancies highlight the need for further research to clarify the clinical boundaries, diagnostic criteria, and treatment implications of central sensitization, particularly in condition-specific contexts such as lumbar disc herniation, while also emphasizing the importance of cautious interpretation and integration of CS within a broader biopsychosocial and mechanism-based framework [23,41,42].

From a diagnostic perspective, the assessment of central sensitization requires a multimodal approach that integrates subjective and objective measures. Instruments such as the Central Sensitization Inventory (CSI), quantitative sensory testing (QST), and conditioned pain modulation (CPM) capture different dimensions of altered pain processing, including symptom burden, sensory hypersensitivity, and impaired descending inhibition [33,35,36,37]. However, these tools are not interchangeable, and their relationships are not fully consistent across studies. For example, recent evidence indicates that associations between CSI scores and QST findings are variable, suggesting that self-reported symptoms and experimentally measured pain processing reflect partially overlapping but distinct constructs [22,36]. Consequently, reliance on a single measure may be insufficient for accurate phenotyping of patients with suspected central sensitization [34].

The therapeutic implications of central sensitization have been widely explored, particularly in the context of chronic musculoskeletal pain. As summarized in Table 2, selected CS-informed interventions target both neurophysiological and psychosocial mechanisms of pain persistence. Systematic reviews and meta-analyses suggest that pain neuroscience education (PNE) may contribute to reductions in pain, disability, catastrophizing, and kinesiophobia, especially when combined with exercise-based interventions. However, the reported effects are not consistently robust across all studies, and some analyses indicate only modest or context-dependent improvements, particularly when PNE is delivered as a standalone intervention rather than combined with active rehabilitation strategies [47,48,51]. Similarly, exercise-based interventions have been shown to modulate central pain processing and improve functional outcomes [41,50], although responses may vary in individuals with elevated sensitization, who often exhibit reduced exercise-induced hypoalgesia [46,50].

The grading system criteria are defined as follows:

- **Grade A** (strong evidence): A preponderance of Level I and/or Level II studies support the recommendation.
- **Grade B** (moderate evidence): A single high-quality randomized controlled trial or a preponderance of Level II studies support the recommendation.
- **Grade C** (weak evidence): A single Level II study supports the recommendation.
- **Grade D** (conflicting or no evidence): Higher-quality studies disagree with respect to their conclusions.

Psychological factors play a critical role in the persistence and modulation of central sensitization. Catastrophizing, kinesiophobia, anxiety, and reduced self-efficacy have been consistently associated with increased pain severity and poorer functional outcomes across chronic pain populations [22,42,70], with recent evidence specifically linking these factors to postoperative recovery in patients with lumbar disk herniation [59]. These factors not only amplify pain perception but also influence behavioral responses, including avoidance and reduced engagement in rehabilitation [61]. Interventions such as cognitive-behavioral therapy and acceptance-based approaches may therefore enhance treatment outcomes by targeting these mechanisms and improving psychological flexibility [46,59,62].

However, integrating these approaches remains challenging because physical rehabilitation providers rarely receive extensive psychological training, while psychologists often lack specialization in pain physiology.

To bridge this interprofessional gap, it is vital to utilize both structured educational frameworks for clinicians and evidence-validated interventions for patients. On one hand, continuing education programs—such as the Integrative Pain Science Institute’s Psychologically Informed Practitioner certification or university-based interprofessional certificates—equip clinicians from diverse backgrounds with the theoretical foundation needed to adopt a biopsychosocial model [71,72]. On the other hand, clinical implementation requires specific programs supported by rigorous peer-reviewed evidence. Most notably, Stanford University’s Empowered Relief program has been validated in randomized controlled trials, demonstrating that a targeted, single-session intervention can be noninferior to standard 8-session cognitive behavioral therapy in reducing pain catastrophizing and improving multiple secondary outcomes in chronic pain populations [73,74]. Distinguishing between clinician-focused training and empirically tested patient interventions fosters a truly integrated, scientifically rigorous approach to chronic pain care [71,72,73,74].

Adjunctive interventions, including dry needling and centrally acting pharmacological agents, may provide additional symptom relief; however, their role should be considered supportive rather than primary. Current evidence suggests that these approaches may reduce symptom burden and facilitate engagement in active rehabilitation [64,65], but they are unlikely to address the underlying multidimensional nature of central sensitization when used in isolation [2,41].

An important limitation of the current evidence base is that much of the research informing central sensitization-oriented management strategies originates from heterogeneous chronic pain populations, including fibromyalgia and other nociplastic conditions, rather than from studies specifically focused on LDH. While these findings may be transferable at a mechanistic level [4,68], their direct applicability to LDH remains uncertain and requires further investigation. This highlights the need for condition-specific research examining the role of central sensitization within clearly defined clinical phenotypes of lumbar disc pathology [27,28].

Fortunately, contemporary translational frameworks and objective diagnostic mapping confirm that LDH presents a distinct clinical entity where peripheral structural drive dynamically interacts with central neuroimmune amplification. Mechanical deformation and the chemical irritation driven by the massive release of pro-inflammatory cytokines (such as TNF-alfa, IL-1 beta, and IL-6) from the herniated nucleus pulposus constitute a potent primary stimulus for peripheral nociceptive firing [21,75]. Rather than remaining restricted to the periphery, this distress initiates a profound neuroimmune crosstalk with the central nervous system. Peripheral neurogenic distress travels via axonal transport or disrupts the blood-spinal cord barrier, activating dorsal horn microglia and astrocytes [21,76]. Once reactive, these central glial cells perpetuate a state of compartmentalized neuroinflammation. The clinical transition into a chronic spinal nociplastic state is characterized by marked neuroinflammatory and glial activation localized directly within the lower back cortical representation of the primary somatosensory and motor cortices (S1/M1), as measured via high-resolution simultaneous PET-fMRI [21,26]. Biochemically, this matches findings from human cerebrospinal fluid analyses, which reveal significantly elevated levels of IL-8 that tightly correlate with clinical vertebral pressure sensitivity and pain intensity, occurring completely independently of systemic blood markers [21,76]. Clinically, when an LDH presentation becomes dominated by central sensitization, traditional peripherally targeted conservative treatments often yield diminishing returns. Once central sensitization is firmly established, the pain experience loses its protective biological function and becomes a pathological condition in itself, rendering standard tissue-focused protocols ineffective [77]. Consequently, while therapeutic interventions are currently extrapolated from broader musculoskeletal cohorts, shifting toward advanced diagnostic frameworks—such as the IASP criteria evaluating widespread pain thresholds and sleep-related symptom burden—is required to accurately identify this specific LDH nociplastic phenotype and implement sensitization-informed rehabilitation. Overall, central sensitization should be understood as a clinically useful, yet not fully delineated, construct that contributes to pain persistence and treatment resistance. Its integration into clinical reasoning may support more comprehensive assessment and individualized rehabilitation strategies, particularly when combined with consideration of structural, neuropathic, psychological, and contextual factors.

## 6. Conclusions

Chronic pain is a multidimensional condition in which symptom persistence is frequently driven by altered central pain processing rather than ongoing tissue pathology. The evidence reviewed here suggests that central sensitization may represent a clinically meaningful framework for understanding chronic musculoskeletal pain, with particular relevance to chronic low back pain and persistent symptoms associated with lumbar disk herniation.

In everyday physiotherapy practice, failure to recognize sensitization-related features may lead to repeated structural interventions with limited long-term benefit. When integrated into clinical practice, targeted interventions such as pain neuroscience education and graded active exercises may improve functional capacity, reduce fear-avoidance patterns, and directly address these underlying central pain mechanisms. Ultimately, this approach informs rehabilitation planning that is more closely aligned with neurobiological mechanisms in patients with chronic low back pain and lumbar disk herniation.

Careful attention is warranted regarding the phenotypic characterization of patients to avoid overgeneralization of central sensitization constructs across heterogeneous chronic pain presentations. Future research should focus on the development and systematic evaluation of structured, central sensitization-informed rehabilitation protocols and their clinical testing in well-characterized patient populations.

## Figures and Tables

**Table 1:** Selected overview of commonly used clinical and research-based instruments for the assessment of central sensitization. This non-exhaustive list highlights core measures targeting altered nociceptive processing and pain modulation, whereas complementary psychological instruments and advanced techniques such as neuroimaging and inflammatory biomarkers provide additional mechanistic insight but are not routinely used for clinical diagnosis.

Tool/Method	Type of Assessment	Primary Construct Assessed	Clinical Relevance	Key References
Central Sensitization Inventory (CSI)	Self-report questionnaire	Self-reported symptom burden associated with central sensitization	Screening and stratification of patients with suspected central sensitization in clinical and research settings	[19,23,32,33,34,36,37]
Quantitative Sensory Testing (QST)	Psychophysical testing	Pain thresholds and sensory hypersensitivity	Objective assessment of altered nociceptive processing and sensory gain	[19,23,32]
Conditioned Pain Modulation (CPM)	Experimental pain modulation paradigm	Efficiency of descending inhibitory pain pathways	Identification of impaired endogenous pain inhibition mechanisms	[23,35]
Pain Catastrophizing Scale (PCS)	Self-report questionnaire	Cognitive-emotional pain amplification (catastrophizing)	Identification of maladaptive pain cognitions associated with increased central sensitization symptom severity and functional impairment	[38,41]
Tampa Scale for Kinesiophobia (TSK)	Self-report questionnaire	Fear of movement and pain-related avoidance behavior	Identification of fear-avoidant behavioral patterns associated with increased central sensitization severity and impaired pain modulation	[25,39,40]
Neuroimaging (fMRI/structural MRI)	Neuroimaging assessment	Altered central pain processing and pain-related brain network activity	Research-level support for altered central pain processing; not routinely used for clinical diagnosis	[23]
Inflammatory biomarkers (e.g., cytokine profiling)	Biochemical assessment	Neuroimmune activity associated with central sensitization	Experimental evidence supporting biological mechanisms underlying central sensitization	[23]

**Table 2:** Summary of selected core and adjunctive interventions used in central sensitization-informed rehabilitation. This non-exhaustive overview focuses on major therapeutic models targeting central pain mechanisms.

Intervention Type	Examples/Delivery	Core Therapeutic Target	Proposed Mechanism Related to CS	Evidence Level	Clinical Relevance	Key References
**Pain Neuroscience Education (PNE)**	Individual/group sessions; metaphors and stories. Preparatory phase before training	Maladaptive pain-related beliefs, catastrophizing, and fear of movement (kinesiophobia)	Pain reconceptualization; deactivating top-down facilitatory pathways	Grade A	Reduces emotional barriers; facilitates engagement in active exercise	[43,45,47,48]
**Exercise-based interventions**	Supervised strengthening and stretching; Water-based AE (13+ weeks)	Impaired central pain modulation, low-pressure pain thresholds (PPT), and movement avoidance	Strengthening descending inhibitory networks (DLPFC, insula); inducing hypoalgesia (EIH)	Grade A	Cornerstone of rehab; helps “overwrite” pain memory via time-contingent approach	[43,56,57,58]
**Dry Needling (manual and electro)**	Manual TrP or Electro-dry needling (EDN). Min. 5 sessions for plastic changes	Peripheral nociceptive input from myofascial trigger points (TrPs) acting as CS mediators	Segmental inhibition; release of opioids/serotonin; suppression of substance P in the spinal dorsal horn	Grade C	Valuable adjunct to reduce CSI scores and facilitate early physical activity	[43,64,65]
**Supportive pharmacological agents**	SNRIs (Duloxetine), Gabapentinoids, TCAs, and Tapentadol	Central pain amplification, sleep disturbances, and imbalanced neurotransmitter systems	Enhancement of descending inhibition; reduction in excitatory neurotransmitter release	Grade B	Restores impaired EIH in early phases, allowing adherence to active protocols	[2,44,46]
**Supportive psychologically informed interventions**	CBT (belief restructuring), ACT, and Mindfulness-based stress reduction	“Cognitive-emotional sensitization,” behavioral avoidance, and low self-efficacy	Reducing brain connectivity in regions associated with pain anticipation; addressing the threat-calm system imbalance	Grade B	Essential for high psychosocial burden; depression strongly predicts CSI scores	[2,20,61,62]

Additional evidence from patients with lumbosacral radiculopathy suggests that electro-dry needling (EDN) may reduce CS-related symptom severity and pain intensity [64]. By providing deeper intramuscular electrical stimulation, EDN has shown greater effectiveness than conventional dry needling in lowering CSI scores, supporting its potential role as an adjunct within multimodal rehabilitation programs for LDH populations [64]. Overall, current clinical guidelines recommend a 6-to-12-week trial of conservative management before surgery is considered, highlighting the value of integrated biopsychosocial care in preventing the transition from acute radiculopathy to persistent centrally mediated pain [10,12]. To resolve reporting inconsistencies, evidence levels for all interventions were standardized to a single grading framework adapted from the clinical practice guidelines [43]. Grades for physical therapy interventions (PNE, exercise, dry needling) were directly extracted from these guidelines. Because centrally acting pharmacological agents and pure psychological interventions fall outside the scope [43], their evidence levels were extrapolated using the same grading criteria based on findings from comprehensive medical and pathophysiological reviews (e.g., [2,44,46]).

## Data Availability

No new data were created or analyzed in this study.
